# Community-based cardiovascular risk assessment using the Cardisio^TM^ AI test: a prospective cohort study

**DOI:** 10.3399/BJGPO.2024.0183

**Published:** 2025-07-16

**Authors:** Simon V Rudland, Nisar H Shah, Alan Nevill

**Affiliations:** 1 Integrated Care Academy, University of Suffolk, Ipswich, UK; 2 Cardiology, Sandwell and West Birmingham Hospitals NHS Trust, Birmingham, UK; 3 University of Wolverhampton Faculty of Education Health and Wellbeing, Walsall, UK

**Keywords:** clinical diagnosis, cardiovascular diseases, inequalities, health inequities, primary health care

## Abstract

**Background:**

Cardiovascular disease (CVD) accounts for significant morbidity and mortality disproportionately affecting hard-to-reach individuals. New technology that enables community testing rather than attending hospital may address health inequalities and facilitate new care pathways.

**Aim:**

To explore whether the Cardisio test, which interprets three-dimensional vectorcardiography activity using a cloud-based artificial intelligence (AI) algorithm, can identify asymptomatic CVD.

**Design & setting:**

Prospective cohort study in three settings: general practice, pharmacy, and a community health centre. Recruitment targeted asymptomatic adults aged ≥18 years, with a QRISK3 score ≥10% or CVD risk factors.

**Method:**

A 10-minute test using five electrodes (four chest, one back). The Cardisio results are classified into red, amber, or green based on the Cardisio test’s perfusion (P), structure (S), and arrhythmia (A) parameters. Pre- and post-test questionnaires provided feedback on participants’ experiences. Results reviewed by a chief investigator ([CI] independent consultant cardiologist) and dealt with according to the study participants’ results and medical profile.

**Results:**

In total, 628 tests were performed, 51% male (*n* = 320), 49% (*n* = 308) female, with a mean age of 54 years (18–75 years). In the opinion of the CI, there was a strong association between one or more Cardisio red test results and referral to cardiology clinic being indicated (*P*<0.001). The test was understood as easy to perform, with an 87.5% recommendation rate among participants (*n* = 492 of the 560).

**Conclusion:**

This simple, near-patient test afforded high-risk hard-to-reach individuals with access to an acceptable test that can facilitate appropriate referral. The automated test does not rely on interpretation of electrocardiogram (ECG) readouts and so is more effective at identifying underlying CVD than a traditional 12-lead ECG.

## How this fits in

This study has found Cardisio to be an acceptable test, which used in the community will help individuals, particularly those who are hard to reach, who have a disproportionate prevalence of cardiovascular disease (CVD). This innovative technology, using cloud-based computing, can facilitate appropriate referral and is good at identifying those requiring further cardiovascular evaluation.

## Introduction

Ischaemic heart disease, associated atrial fibrillation, and acute ischaemic events account for significant morbidity and mortality. Alongside the individual and social impact, a large burden of care is placed on the NHS.^1–4^ Often these events disproportionately affect hard-to-reach members of society who may have a predisposition because of their ethnicity and persisting socioeconomic inequalities.^5^ Recently concerns regarding the large numbers of people with undiagnosed heart failure have been voiced in national media.^6^ New technology that enables accessible near-patient testing, and empowers clinical decision making in primary care, may begin to address these health inequalities.^1,7^ A simple test performed in a community setting that can reliably identify or exclude abnormalities of the heart may identify asymptomatic disease while reducing unnecessary referral to hospital-based cardiologist.

The Cardisio test captures three-dimensional vectorcardiography activity (the recording method for cardiac electrical activity traditionally in two dimensions), through the placement of an electrode on the participant’s back. The large amount of data generated is interpreted using a cloud-based AI assistant algorithm (the cloud’s computing power can be accessed in real time from any location). The Cardisio web services uses AI-based tools. The machine learning model combines five neural networks that evaluate different parameters averaged over all the heart beats measured for each patient. The test usually takes 10 minutes^8^ to perform and is able to detect changes in perfusion (P) structure (S), and identify arrhythmias (A).^9–11^ The Cardisio service is a Class I CE-marked medical device. The company is accredited to ISO 13485:2016 quality management system for medical devices. The technology has been used extensively in Europe and can identify coronary artery disease at rest with, for female patients, a sensitivity of 90·2±4·2% (male: 97·2±3·1%), specificity of 74·4±9·8% (male: 76·1±8·5%), and overall accuracy of 82·5±6·4% (male: 90·7±3·3%).^12,13^ The test results provide a red, amber, or green status for each of the measured values P, S, and A. The national NHS England Core20PLUS5 action plan hopes to reduce healthcare inequalities at both national and system levels.^14^

This Small Business Research Initiative (SBRI)-supported study set out to explore whether the Cardisio test could be used in conjunction with existing NHS community-based cardiovascular disease (CVD) prevention strategies for early detection of CVD. We delivered the study in ethnically diverse boroughs of Sandwell (48% of residents are from Black and minority ethnic communities) and Dudley (4.6% Pakistani, and 2.4% Indian).^15^ We gathered and evaluated quantitative and qualitative data during the study.

## Method

### Study population

**Figure 1.**
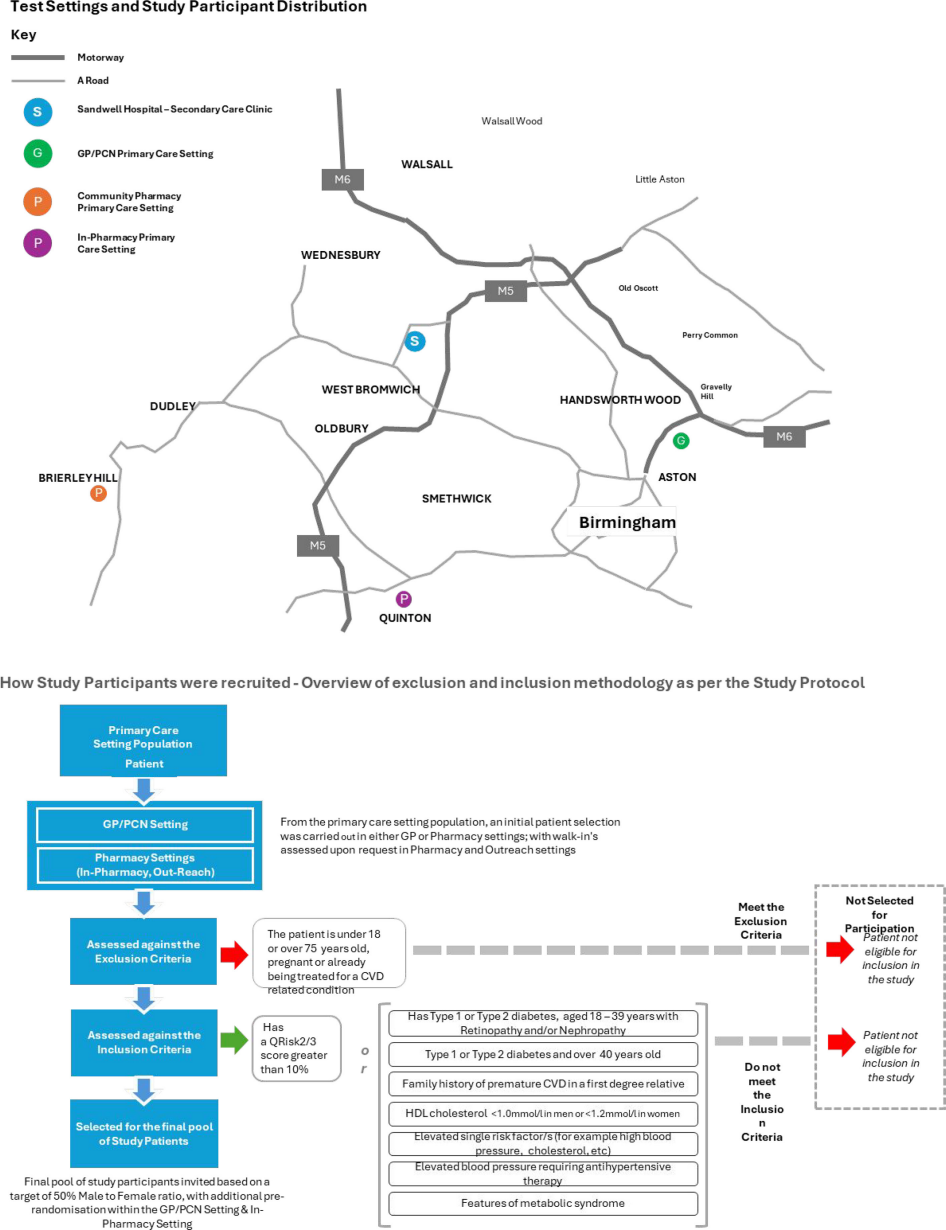
Study locations and the recruitment method. CVD = cardiovascular disease. HDL = High-density lipoprotein. PCN = primary care network

This prospective cohort study was based in three settings: a general practice, a pharmacy; and a community health centre. Recruitment through invitation targeted asymptomatic adults aged ≥18 years, with no prior diagnosis of severe heart conditions but who had a QRISK3 score ≥10% or other CVD risk factors. Participants were recruited within the general practice or pharmacy setting where there was access to their clinical record or prescription record indicating a risk of CVD. Figure 1 shows the test area and the methodology for selecting and inviting potential study participants. Although not a randomised trial, we sought to adopt the Consolidated Standards of Reporting Trials (CONSORT) 2010 minimum guidelines for randomised studies involving AI.^16^ We have set out to adhere to the Strengthening the Reporting of Observational studies in Epidemiology (STROBE) checklist for cohort studies.^17^ Participant enrolment began on 14 August 2023 and ended on 24 February 2024; the final appointment with the CI took place on 21 March 2024. Data analysis was completed on 21 August 2024.

### Testing

Testing involved the placement of four chest electrodes and one on the back. Heart activity was captured using the electrodes and a small gateway device that connects via a laptop or PC to a cloud-based web service. The test is completely automated and does not rely on interpretation of an electrocardiogram (ECG) readout, with results classified into red, amber, or green based on the Cardisio test’s perfusion (P), structure (S), and arrhythmia (A) parameters. A power calculation using a prevalence of 5% and precision of 1.5% suggested a sample size of 811 would correspond with a 95% confidence interval.^18^ A total of 692 participants registered to take part. By the study’s conclusion, 628 tests had been conducted. Ethical approval was granted (REC reference 23/PR/0740) and we engaged with patients while designing the study. This included ensuring sex balance was achieved, how tests were facilitated, and that hard-to-reach communities were served using community outreach.

### Data acquisition

Once a participant had been tested they were allocated a unique study reference number in order to secure anonymity of their data. No personal participant data were shared with the cloud-based AI algorithm. The quantitative dataset included participant age; height; weight; postcode; GP; name and address; reason for inclusion in the study (which risk factors triggered invitation to participate); date and time of test; and test administrator. It also included the Cardisio test reference number; P, S, and A parameters and the red, amber, or green classification; and notes added by test administrator, chief investigator (CI, consultant cardiologist), and Cardisio team capturing next steps for the participant. The participants completed the questionnaire immediately pre- and post-test wherever possible (Annex 1). The responses to the questions provided feedback from study participants of their experience of the administration of a Cardisio test. These data were gathered in a qualitative dataset. The test sites were incentivised to carry out this additional work through a payment to cover the costs of each completed test.

Results with a minimum of one amber or one red test in any of the test parameters (P, S, and A) were subject to a desktop review by the CI to decide whether further investigative action was required. The CI was able to review the individual participant’s clinical risk factors along with a 12-lead ECG generated by Cardisio. Where judged appropriate, selected individuals were seen by the CI in their cardiac outpatient department.

### Statistical analysis

The χ^2^ test was used to explore the strength of association between those tests judged abnormal or normal by the Cardisio test and the CI.

## Results

### Quantitative results

**Figure 2.**
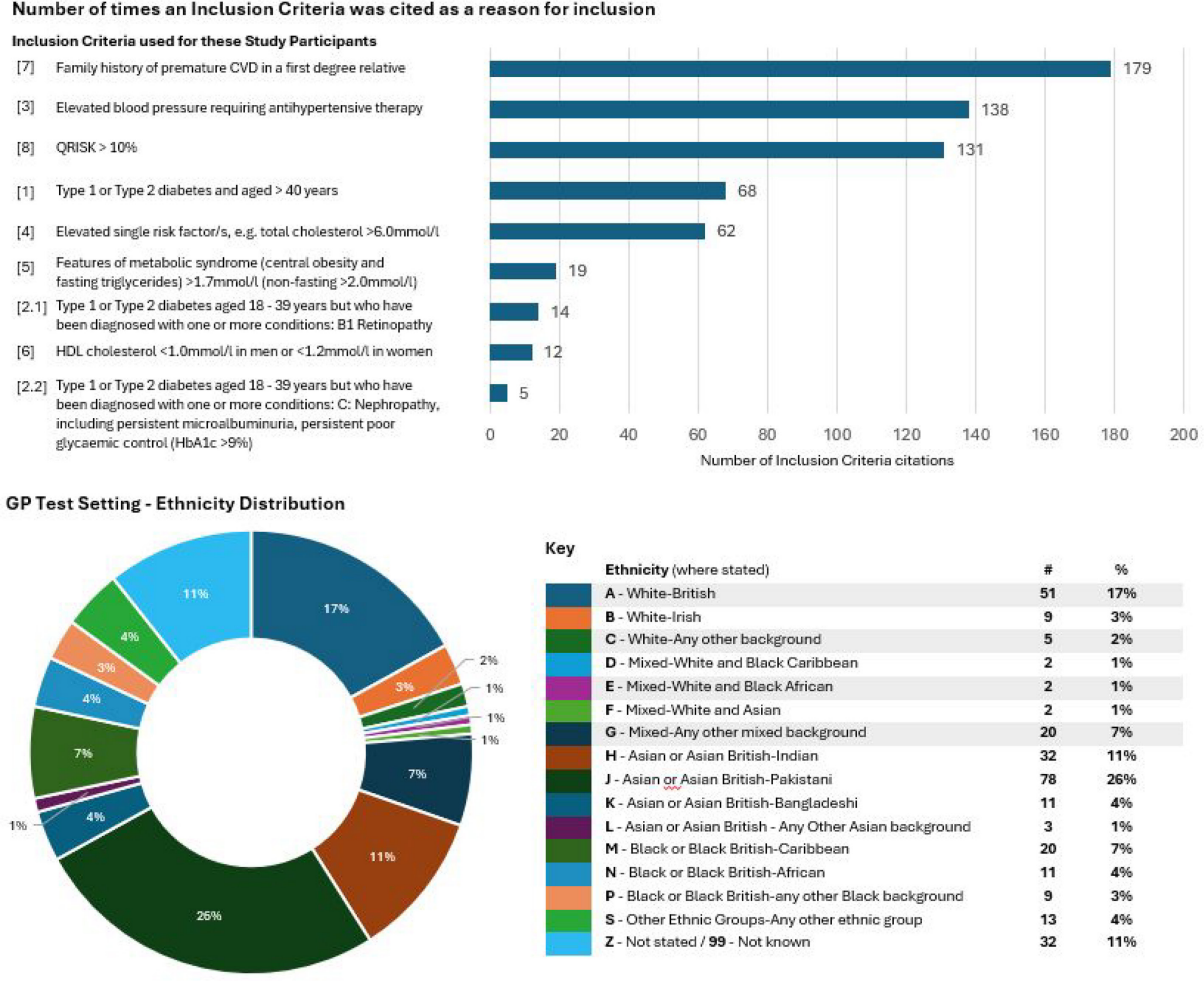
Inclusion criteria and ethnicity of participants. GP test setting *N* = 300. . CVD = cardiovascular disease. HDL = High-density lipoprotein

By the end of the study period, 628 tests had been performed; 51% male (*n* = 320) and 49% (*n* = 308) female with a mean age of 54 years (18–75 years). Inclusion criteria are summarised in Figure 2 for all participants, along with ethnicity details for those who attended the general practice setting. Difficulty in recruiting limited the size of our sample set, consequently reducing the statistical power of the study but not below the significance threshold. Fifty-four per cent (*n* = 340) of tests had a minimum of one amber or one red test result, 46% (*n* = 288) of tests were all green. Following desktop review, the CI identified that 24% (*n* = 150) of the participants with an abnormal result may require an outpatient follow-up appointment. The CI reviewed a random selection of 35 participants who had a green result for all three parameters and judged that these results were true negatives.

**Figure 3.**
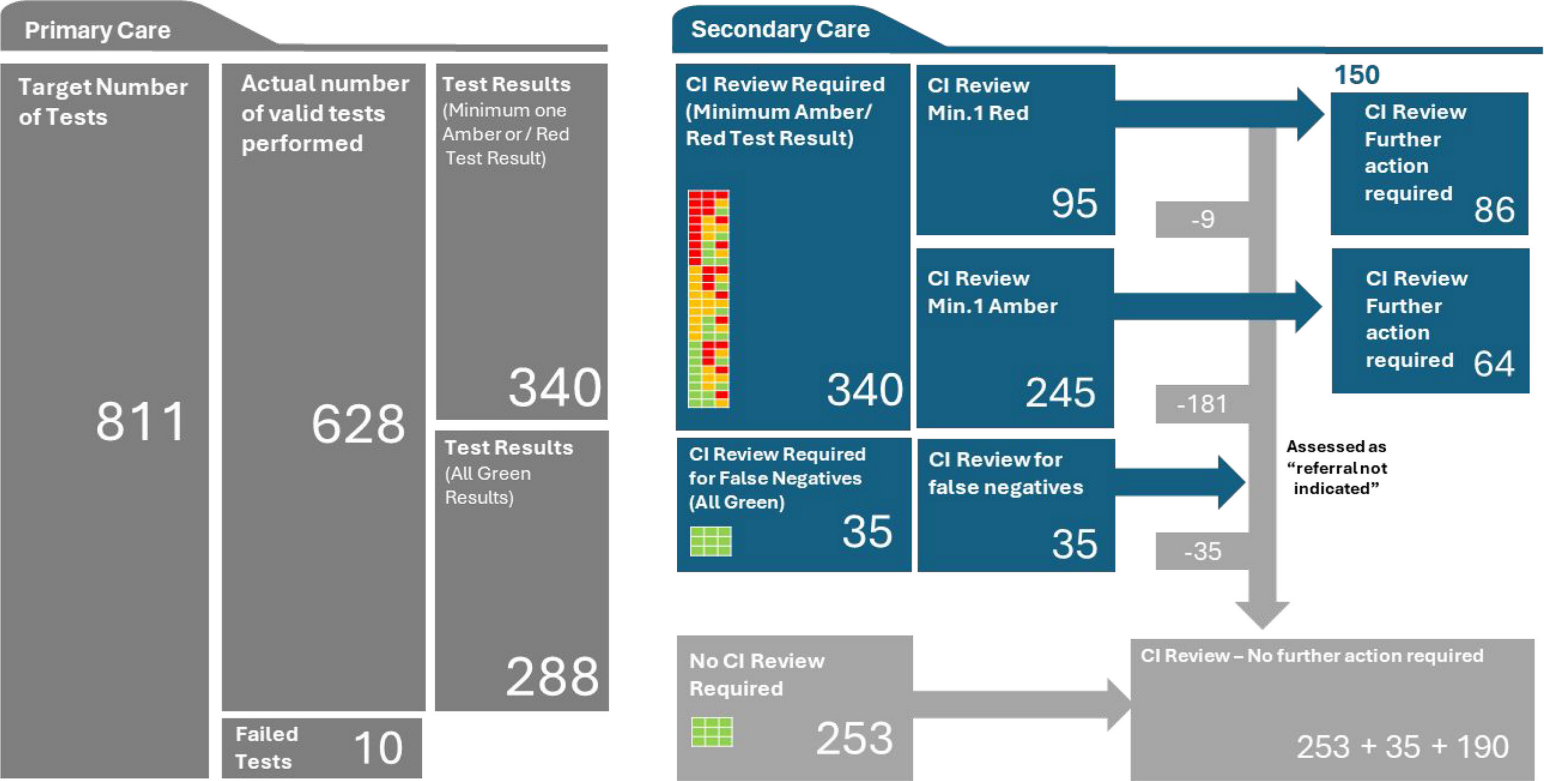
Summary of quantitative results. CI = chief investigator

**Figure 4.**
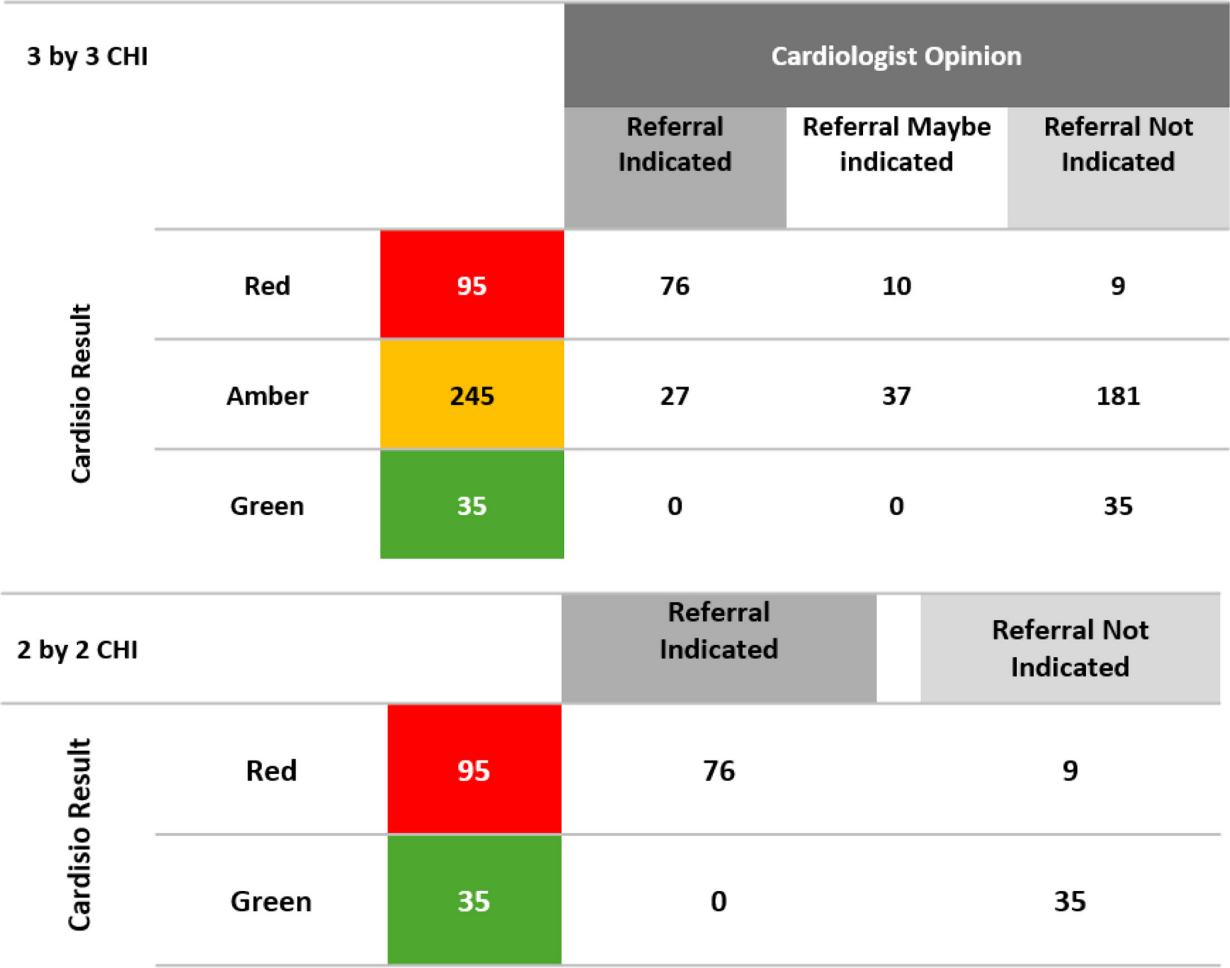
Summary of qualitative result indicating the relationship between chief investigator (CI) opinion and the Cardisio results

The study findings are summarised in Figures 3 and 4. There was a strong association between the Cardisio test result of one or more red result and the CI assessment that referral to a secondary care cardiology clinic was or maybe indicated (*P*<0.001). The presence of a negative Cardisio test was strongly associated with the CI decision that no further cardiovascular intervention was required (*P*<0.001). Seventy-four per cent (*n* = 181) of the amber test result group were judged not to require a referral to secondary care cardiology clinic. The Cardisio test had a positive predictive value (PPV) of 80.0% and negative predictive value (NPV) of 90.4% (prevalence of 5%, *Z* = 1.96, precision 1.7% *n* = 628). In the opinion of the CI, there were *n* = 9/225 (4.0%) false positives and *n* = 0/103 (0%) false negatives.

The (3 by 3) χ^2^ test of independence (χ^2^ = 192.8 with 4 degrees of freedom) and χ^2^ test of linear trend (χ^2^ = 159.1 with 1 degree of freedom) revealed a strong relationship between the CI opinion and the Cardisio results (both *P*<0.001). Focusing on just the red and green Cardisio results and their association with the CI opinion (referral indicated versus not indicated), we observed an even stronger association, as seen in the 2 by 2 χ^2^ table (Figure 4).

The reduced (2 by 2) χ^2^ test of independence (χ^2^ = 85.3 with 1 degrees of freedom) and χ^2^ test of linear trend (χ^2^ = 81.5 with 1 degree of freedom) reveals an even stronger relationship between the CI opinion and the Cardisio results (both *P*<0.001).

**Figure 5.**
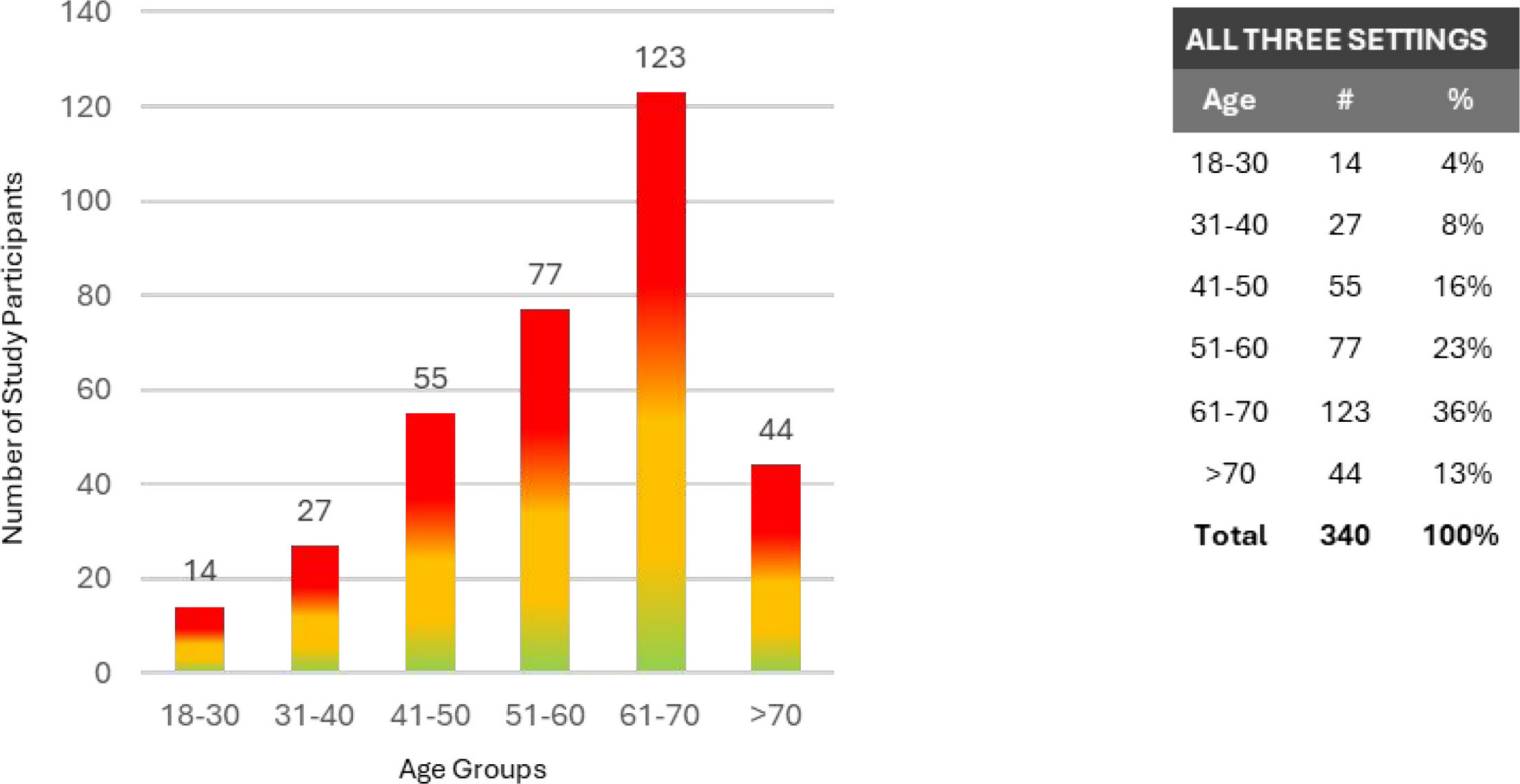
Age profile and frequency of positive tests in combined site

The corresponding Kappa statistic for the above (2 by 2) table is 0.831, confirming an excellent measure of agreement between the CI opinion and the Cardisio results. Thirty-five of all green test results were selected at random and subjected to desktop evaluation by the CI. These negative results were identified as reflecting true negatives. If we assume those 35 selections to be representative, the Cardisio test has a sensitivity of 73.8% and specificity of 94.4%. The test settings revealed a broadly similar age profile with the predominance of at least one amber and one red result found between the ages of 61 years and 70 years (Figure 5). Less than 2% of the conducted tests (*n* = 10) failed as a consequence of technical problems.

### Qualitative results

Ninety per cent (general practice *n* = 303, in pharmacy *n* = 162, community pharmacy *n* = 103) of participants completed their pre-test questionnaires and 90% (general practice *n* = 302, in pharmacy *n* = 152, community pharmacy *n* = 113) completed their post-test questionnaires. The majority of participants agreed to take the test because they had been asked by their pharmacist or GP (35% of those who answered, *n* = 185 of 533), or because they had concerns about the heart health (34% of those who answered, *n* = 189 of 560) or because there was a family history of CVD (26% of those who answered, *n* = 148 of 560). The majority of participants had not previously been diagnosed with a heart condition (89% of those who answered, *n* = 502 of 567). The study pamphlet was welcoming and effective in explaining the Cardisio test (96%, *n* = 414 of 430), although a large minority had not received any information before joining the study (18%, *n* = 100 of 544). The post-test feedback reports were predominantly positive in terms of test experience (99%, *n* = 563 of 567 of those who answered), test administrator’s preparedness (99%, *n* = 552 of 554 of those who answered), and documentation quality (99% , *n*=552 of 560 who answered). This suggests a successful approach to engagement with the community and study participants. Test teams were able to provide effective explanations of the post-test process across all of the settings (98%, *n* = 558 0f 567 who answered). There were high satisfaction ratings; 76% (*n* = 417 of 552 who answered) were very satisfied and 22% (*n* = 124 of the 552 who answered) were satisfied with the Cardisio test experience. Overall, 88% would definitely recommend the Cardisio test to others (*n* = 492 of the 560 who answered).

## Discussion

### Summary

We found that the Cardisio test is easy to learn for the administrators and has a high level of satisfaction and acceptability from participants. The test has high PPV (80.0%) and NPV (90.4%) and a sensitivity of 73.8% and specificity of 94.4%. The test aligns with the NHS England Core20PLUS5 goals. In the opinion of the CI, a red result is a strong predictor of underlying CVD that requires further secondary care assessment. A green result is highly reassuring of ’no underlying CVD’ in the real-world setting. This simple, near-patient test afforded high-risk hard-to-reach individuals with access to an acceptable test that can facilitate appropriate referral. The automated test does not rely on interpretation of ECG readouts and so is more effective at identifying underlying CVD than a traditional 12-lead ECG. In the opinion of the CI, a red result was strongly correlated with underlying pathology while a green result made this very unlikely (*P*<0.001), without the need for trace interpretation.

### Strengths and limitations

This is the first time a new ECG technology has been used in a UK community setting. We enabled a sensitive and specific near-patient test to be administered to an at-risk population who might otherwise have not had access to this type of care. The absence of false negatives suggests participants were not given false reassurance regarding their investigations. The 4% (*n* = 9/225) false positives may result in unwarranted patient anxiety and needless additional investigations. Many patients in primary care have not had QRisk calculated,^19^ which is why we included other possible predictors of CVD (Figure 1). Participants were reached in a variety of community settings, with less than 2% of tests failing. An important minority of asymptomatic participants 21% (*n* = 134) were seen in cardiology as outpatients and ultimately 13% (*n* = 80) were referred for further cardiology investigations. We believe that these data indicate that the Cardisio test has the potential to address part of the national NHS England Core20PLUS5 action plan, and improve care delivered to hard-to-reach members of our communities who are at risk of CVD. The opportunity to confidently exclude CVD abnormalities with this test may reduce the number of referrals made to cardiology outpatient clinics. Together with offering a near to patient test the carbon footprint of the patient journey may be reduced.^20^

This study was funded by the SBRI and Cardisio. The datasets were administered independently of Cardisio and the three test centres, with patient anonymity being preserved. Each of the test teams functioned independently of each other. The CI had access to the full dataset and is independent of Cardisio working in an NHS setting within the UK. The amber test results reflect participants who need additional clinical consideration. In our real-world setting, many had a simple conduction abnormality or minor rhythm disturbance that was clinically insignificant. As more patient data are analysed the AI-driven decision-making algorithm will evolve enabling dynamic adjustments, improving the accuracy of this test, and reduce the number of amber results. This observational study has several limitations. Participants were recruited through invitation, possibly introducing selection bias. The CI was responsible for selecting patients to be reviewed in their own clinic, which introduces observer bias into the decision-making pathway. The AI algorithms were developed in Germany in settings that do not reflect the ethnic diversity of our study population. Economic and ethnic bias in AI algorithms can exacerbate health inequalities.^21^ Potential bias in the Cardisio AI-driven decision-making algorithm will have less impact as more diverse groups are investigated. To help reduce bias the machine learning model is a combination of five neural networks calculating parameters for each heart beat independently of patient sex or age. The network is trained using controlled patient datasets and supervised parameters to improve the ability to correctly identify true positives and true negatives. The study did not achieve its original recruitment target of 811 participants. Research carried out in community settings is very challenging owing to competing interest. During this study, the administration of flu vaccinations impacted on recruitment. Despite this, we achieved statistically significant result. The timeframe and funding for this study has not enabled us to include the long-term cardiovascular outcome data from patients referred for further investigation by the CI.

### Comparison with existing literature

The ECG is a frequently deployed investigation in primary care, although accurate interpretation of the ECG trace is difficult. Begg *et al* ^22^ observed that primary care clinicians interpreted abnormal ECG as normal in 23% of the responders, compared with just 3% of cardiologists. Sahota and Taggar^23^ recognise that primary care clinicians need help in improving their interpretation of ECGs, while an Australian study suggested the interpretive function of the ECG machine should viewed with ‘extreme caution’.^24^ The Cardisio test has three outputs: red, amber, or green in three modalities: perfusion, rhythm, and structure. This potentially simplifies patient assessment in primary care and enhances diagnostic accuracy in a setting where there is often a very high level of uncertainty and anxiety about missing underlying significant pathology.^25,26^ Technologies, such as the Apple Watch and KardiaMobile,^27^ enable individuals to assess their own rhythm or facilitated near-patient ECG acquisition.^28^ These devices are only interpreting rate and rhythm. Cardio-HART ^29^ is a non-invasive patient test, as yet not evaluated in a real-world setting, requiring a more complex participant set-up than the Cardisio test.

### Implications for research and practice

Timely access to easily administered cost-effective investigations that clearly identify individuals in need of onward referral provide powerful opportunities to disrupt and improve on current referral pathways. A more precise test will enable patients with proven underlying CVD to be referred into secondary care, possibly helping to reduce waiting lists. We hope that validation and applicability of this study’s finding can be explored through the use of the Cardisio test in more primary care settings. Further clinical and economic evaluation will establish whether its use better identifies patients in need of secondary care investigation, while enabling those at low or no cardiovascular risk to be managed in primary care.

## Data

The data generated by the testing complies with the NHS data protection requirements. There was no third-party access to the study participant data, and the Secondary Care team managed the central data. Cardisio^TM^ only had access to anonymised, non-identifiable data to support the testing. The dataset relied on in this article is available from the corresponding author on reasonable request.
